# Extracellular SOD-Derived H_2_O_2_ Promotes VEGF Signaling in Caveolae/Lipid Rafts and Post-Ischemic Angiogenesis in Mice

**DOI:** 10.1371/journal.pone.0010189

**Published:** 2010-04-21

**Authors:** Jin Oshikawa, Norifumi Urao, Ha Won Kim, Nihal Kaplan, Masooma Razvi, Ronald McKinney, Leslie B. Poole, Tohru Fukai, Masuko Ushio-Fukai

**Affiliations:** 1 Center for Lung and Vascular Biology, Department of Pharmacology, University of Illinois at Chicago, Chicago, Illinois, United States of America; 2 Department of Medicine and Pharmacology, University of Illinois at Chicago, Chicago, Illinois, United States of America; 3 Center for Cardiovascular Research, University of Illinois at Chicago, Chicago, Illinois, United States of America; 4 Department of Biochemistry, Wake Forest University School of Medicine, Winston-Salem, North Carolina, United States of America; Louisiana State University, United States of America

## Abstract

Reactive oxygen species (ROS), in particular, H_2_O_2_, is essential for full activation of VEGF receptor2 (VEGFR2) signaling involved in endothelial cell (EC) proliferation and migration. Extracellular superoxide dismutase (ecSOD) is a major secreted extracellular enzyme that catalyzes the dismutation of superoxide to H_2_O_2_, and anchors to EC surface through heparin-binding domain (HBD). Mice lacking ecSOD show impaired postnatal angiogenesis. However, it is unknown whether ecSOD-derived H_2_O_2_ regulates VEGF signaling. Here we show that gene transfer of ecSOD, but not ecSOD lacking HBD (ecSOD-ΔHBD), increases H_2_O_2_ levels in adductor muscle of mice, and promotes angiogenesis after hindlimb ischemia. Mice lacking ecSOD show reduction of H_2_O_2_ in non-ischemic and ischemic limbs. *In vitro*, overexpression of ecSOD, but not ecSOD-ΔHBD, in cultured medium in ECs enhances VEGF-induced tyrosine phosphorylation of VEGFR2 (VEGFR2-pY), which is prevented by short-term pretreatment with catalase that scavenges extracellular H_2_O_2_. Either exogenous H_2_O_2_ (<500 µM), which is diffusible, or nitric oxide donor has no effect on VEGF-induced VEGFR2-pY. These suggest that ecSOD binding to ECs via HBD is required for localized generation of extracellular H_2_O_2_ to regulate VEGFR2-pY. Mechanistically, VEGF-induced VEGFR2-pY in caveolae/lipid rafts, but non-lipid rafts, is enhanced by ecSOD, which localizes at lipid rafts via HBD. One of the targets of ROS is protein tyrosine phosphatases (PTPs). ecSOD induces oxidation and inactivation of both PTP1B and DEP1, which negatively regulates VEGFR2-pY, in caveolae/lipid rafts, but not non-lipid rafts. Disruption of caveolae/lipid rafts, or PTPs inhibitor orthovanadate, or siRNAs for PTP1B and DEP1 enhances VEGF-induced VEGFR2-pY, which prevents ecSOD-induced effect. Functionally, ecSOD promotes VEGF-stimulated EC migration and proliferation. In summary, extracellular H_2_O_2_ generated by ecSOD localized at caveolae/lipid rafts via HBD promotes VEGFR2 signaling via oxidative inactivation of PTPs in these microdomains. Thus, ecSOD is a potential therapeutic target for angiogenesis-dependent cardiovascular diseases.

## Introduction

Angiogenesis is involved in physiological process such as development and wound healing as well as pathophysiologies such as ischemic heart and limb diseases, atherosclerosis and cancer. In endothelial cells (ECs), vascular endothelial growth factor (VEGF) induces angiogenesis by stimulating EC proliferation and migration primarily through the VEGF receptor type2 (VEGFR2, KDR/Flk1) [1]. VEGF binding initiates autophosphorylation of VEGFR2, which is followed by activation of diverse downstream signaling events linked to angiogenesis in ECs [2], [3]. Reactive oxygen species (ROS), in particular H_2_O_2_, function as key signaling molecules to mediate various biological responses including angiogenesis. Reversible oxidative inactivation of reactive (low pKa) cysteinyl residues (Cys-SH) at active sites in protein tyrosine phosphatases (PTPs) is important mechanism by which ROS stimulate tyrosine phosphorylation-dependent redox signaling events [4], [5]. We and others reported that ROS derived from NADPH oxidase play an important role in VEGFR2-mediated signaling linked to EC migration and proliferation [6], [7], [8] as well as post-ischemic angiogenesis *in vivo* [9], [10]. Evidence reveals that extracellular redox state regulates intracellular signaling [11] or tumor growth [12] by modulating plasma membrane-associated proteins. Exogenous H_2_O_2_ induces expression of both VEGF and VEGFR2 [13] and pro-angiogenic responses in ECs [8]. Since H_2_O_2_ is diffusible molecule, we have posited that generating extracellular H_2_O_2_ at site of VEGFR2 activation in the specific subcellular compartment is important therapeutic approach to promote VEGF signaling linked to angiogenesis.

Extracellular superoxide dismutase (ecSOD, SOD3) is the major SOD in the vascular extracellular space that catalyzes dismutation of superoxide anion (O_2_^−^) to H_2_O_2_ [14]. ecSOD is highly expressed in blood vessels and lung, and synthesized and secreted by a variety of fibroblasts [15]. Importantly, ecSOD is anchored to EC surface via binding with heparan sulfate proteoglycans (HSPGs) through a heparin-binding domain (HBD) [16]. *In vivo*, ecSOD has been implicated in protecting endothelial function in various cardiovascular diseases by controlling the levels of extracellular O_2_^−^ and nitric oxide (NO) bioactivity in the vasculature [14]. We showed that ecSOD expression is markedly increased in ischemic tissues in response to hindlimb ischemia, and that mice lacking ecSOD show impaired post-ischemic neovascularization [17]. However, a role of ecSOD-derived extracellular H_2_O_2_ in VEGF signaling and postnatal angiogenesis remains unknown.

Caveolae and lipid rafts are cholesterol- and sphingolipid-rich plasma membrane microdomains, in which multiple signaling molecules and receptors are assembled to provide the molecular proximity for rapid, efficient, and specific activation of downstream signaling [18]. VEGF-induced VEGFR2 autophosphorylation initially occurs in caveolin-enriched lipid rafts [19], [20] where NADPH oxidase subunits [21] are localized in ECs. Several PTPs, which are targets of ROS, as described above, are also found in caveolae/lipid rafts in non-vascular systems [22], [23]. Among PTPs, PTP1B [24] and density-enhanced phosphatase-1 (DEP-1)/CD148 [25] are major endogenous negative regulator for VEGFR2 tyrosine phosphorylation in ECs. However, their presence in lipid rafts and oxidation in ECs have not been demonstrated. VEGFR2 signaling is also regulated by HSPGs [26], [27], and some core proteins of HSPGs localize in caveolae/lipid rafts [28], [29]. Given that ecSOD binds to HSPGs via HBD, we hypothesized that ecSOD-derived extracellular H_2_O_2_ may locally regulate VEGFR2 signaling to promote angiogenesis.

Here we demonstrate that gene transfer of ecSOD increases H_2_O_2_ levels in adductor muscle, and promotes angiogenesis after hindlimb ischemia, in a HBD-dependent manner. Mice lacking ecSOD show reduction of H_2_O_2_ production in both non-ischemic and ischemic limbs. *In vitro*, H_2_O_2_ generated extracellularly by ecSOD anchored to ECs surface via HBD enhances VEGF-induced VEGFR2 autophosphorylation in caveolin-enriched lipid rafts, but not in non-lipid rafts. HBD of ecSOD is required for localization of ecSOD at plasma membrane lipid rafts where VEGFR2 and PTP1B/DEP-1 are found. ecSOD promotes oxidative inactivation of PTP1B and DEP1 in caveolae/lipid rafts as well as VEGF-induced EC migration and proliferation. These findings suggest that localization of ecSOD in caveolae/lipid rafts via HBD can serve as an important mechanism by which ecSOD-derived extracellular H_2_O_2_ efficiently promotes VEGFR2 signaling in ECs and postnatal angiogenesis.

## Methods

### Animals

Study protocols were approved by the Animal Care and Institutional Biosafety Committee of University of Illinois at Chicago (ACC: 09-066).

### Materials

Adenovirus expressing wild-type human ecSOD (Ad.ecSOD) and human ecSOD lacking heparin binding domain (Ad.ecSOD-ΔHBD) were from adenovirus core at University of Iowa [16]. Anti-human ecSOD antibody was kindly provided by Dr. David Harrison at Emory University [30]. Anti-mouse ecSOD antibody has been described previously [31]. Antibodies to VEGFR2, phosphotyrosine (pY99) and paxillin were from Santa Cruz. Antibodies to phospho-VEGFR2 (pY1175) were from Cell Signaling. Anti-PTP1B antibody was from Calbiochem. Anti-caveolin-1 antibody was from BD Biosciences. Anti-DEP-1 antibody was from R&D systems. Human recombinant VEGF165 was from R&D Systems. Oligofectamine, and Opti-MEMI Reduced-Serum Medium, were from Invitrogen Corp. Catalase was from Calbiochem. Other materials were purchased from Sigma.

### Cell Culture

Human umbilical vein ECs (HUVECs) were grown in endothelial basal medium2 (EBM2, Clonetics) containing 5% fetal bovine serum (FBS) as described [7].

### Immunoprecipitation and Immunoblotting

Growth-arrested HUVECs were stimulated with VEGF (20 ng/ml) and cells were lysed in lysis buffer, pH 7.4 (in mM) 50 HEPES, 5 EDTA, 100 NaCl), 1% Triton X-100, protease inhibitors (10 µg/ml aprotinin, 1 mmol/L phenylmethylsulfonyl fluoride, 10 µg/ml leupeptin) and phosphatase inhibitors ((in mmol/L) 50 sodium fluoride, 1 sodium orthovanadate, 10 sodium pyrophosphate). Cell lysates were used for immunoprecipitation and immunoblotting, as described previously [32].

### Adenovirus Transduction

HUVECs were incubated with 5 multiples of infection (MOI) of either Ad.ecSOD or Ad.ecSOD-ΔHBD or Ad.LacZ (control) in 5% FBS containing culture medium for 24 hr, followed by incubation in 0.5% FBS containing culture medium without virus for 24 hr before experiments, as we described previously [20]. For experiments using conditioned medium, 0.5% FBS containing culture medium obtained from ECs infected with Ad.LacZ or Ad.ecSOD for 24 hr was applied to other HUVECs without infection.

### H_2_O_2_ measurement

H_2_O_2_ production was detected by incubating the cells with 20 µM 5-(and-6)-chloromethyl-2′,7′-dichlorodihydrofluorescein diacetate, acetyl ester (CM-H_2_DCFDA, Invitrogen) for 15 min at 37°C and observed by confocal microscopy using same exposure condition in each experiment. Relative DCF-DA fluorescence intensity was recorded and analyzed using ImageJ as we reported previously [32].

### Superoxide Dismutase Activity Assays

To isolate ecSOD from conditioned media, Con A-Sepharose chromatography (Pharmacia Biotech) was used, as described previously [33]. Unlike Cu/Zn SOD and Mn SOD, the glycoprotein in ecSOD binds to the lectin concanavalin A. Conditioned media were applied to a Con A-Sepharose column equilibrated with 50 mM potassium phosphate buffer (pH 7.4) in 120 mM NaCl. ecSOD fraction was eluted with 150 mM α-methyl mannoside in 50 mM potassium phosphate buffer (pH 7.4). SOD activity was measured in 50 mM phosphate buffer by inhibition of the reduction of cytochrome C (50 µM) by superoxide generated by xantine oxidase (0.01 U/ml) at pH 7.4.

### Amplex Red assay

H_2_O_2_ formation in non-ischemic and ischemic adductor muscle (1–2 mg) was measured by Amplex Red assay, which predominantly detects extracellular H_2_O_2_, according to manufacturer's instruction (Invitrogen). The values were standardized with tissue weights.

### Sucrose Gradient Fractionation

Caveolae/lipid rafts fractions were separated, as described previously [34]. Briefly, HUVECs (5.0×10^7^ cells) or mouse lung (400 mg) were homogenized in a solution containing 0.5 M sodium carbonate (pH 11), 1 mM sodium orthovanadate and protease inhibitors. The homogenates were adjusted to 45% sucrose by adding 90% sucrose in a buffer containing 25 mM Mes (pH 6.5) and 0.15 M NaCl. A 5–35% discontinuous sucrose gradient was formed above and centrifuged at 39,000 rpm for 16–20 hrs in a Beckman SW-40Ti rotor. From the top of the tube, 13 fractions were collected, and an equal volume from each fraction was subjected to immunoblotting. To quantify the protein expression levels in caveolae/lipid rafts and non-caveolae/lipid rafts fractions, equal volume of fractions 4–6 were combined for caveolae/lipid rafts and fractions 9–13 were combined for non-caveolae/lipid rafts. In some experiments, HUVECs were lysed in 25 mM Mes (pH 6.5), 0.15 M sodium orthovanadate, 0.1% Triton X-100 and protease inhibitors, and used for PTPs activity and oxidation assays.

### siRNA Transfection

RNA oligonucleotides were obtained from Sigma or Ambion. siRNA against PTP1B is described previously and siRNA against DEP-1 is from Silencer® Select Pre-designed siRNA (Ambion). HUVECs were grown to 40% confluence in 100 mm dishes and transfected with 10 nM siRNA using Oligofectamine (Invitrogen), as described previously [35]. Cells were used for experiments at 48 hr after transfection.

### PTP Activity Assay

Specific PTP1B and DEP-1 PTPs activities were measured by the hydrolysis of p-nitrophenyl phosphate (pNPP; Sigma). Briefly, PTP1B and DEP1 immunoprecipitates from sucrose gradient-fractionated samples were incubated in a final volume of 100 µl at 37°C for 30 min in reaction buffer containing 10 mM pNPP. The reaction was stopped by the addition of 200 µl of 5 M NaOH, and the absorption was determined at 410 nm [24].

### Sulfenic Acid (Cys-SOH) labeling

For the labeling of Cys-SOH in proteins, HUVEC were lysed in de-oxygenized ice-cold lysis buffer containing 0.1 mM Cys-SOH trapping reagent, [36], [37], 200 U/ml Catalase, 100 µM DTPA, 5 mM iodoacetamide. In order to affinity enrich for biotin labeled proteins modified by the Cys-SOH probe, lysates were incubated overnight with Streptavidin beads (Thermoscientific), and precipitated samples were subjected for immunoblotting.

### Cell Proliferation Assay

HUVECs (10^5^ cells) were seeded in 6-well plates in EBM2 containing 5% FBS overnight, and incubated in EBM2 containing 0.5% FBS for 24 hours and then incubated with or without stimulants in EBM containing 0.2% FBS for 48 hours. After trypsinization, the cell number was determined by counting with a hemocytometer as described before [7].

### Modified Boyden Chamber Migration Assay

Migration assays using a Modified Boyden Chamber method were conducted in 24-well transwell chambers as described previously [7].

### Mouse Ischemic Hindlimb Model

Female C57BL/6J mice (8–9 weeks of age) were obtained from The Jackson Laboratory. The ecSOD-deficient mice in a C57Blk/6 background were described previously [17]. The superficial femoral artery was ligated proximally and distally with 5–0 silk ligatures, and excised. After surgery, adenovirus expressing human ecSOD or ecSOD-ΔHBD or LacZ was injected into adductor muscle at 1×10^9^ pfu. To measure hind limb blood flow we used a laser Doppler blood flow (LDBF) analyzer (Lisca AB, Sweden) as described previously [9]. At 7 days after ischemia, thigh adductor muscles in ischemic hindlimbs were used for immunohistochemistry as described previously [9], [10], [20].

### Statistical Analysis

Results are expressed as mean ± S.E. Statistical significance was assessed by Student's paired two-tailed t-test or analysis of variance on untransformed data, followed by comparison of group averages by contrast analysis using the Super ANOVA statistical program (Abacus Concepts, Berkeley, CA). A p value of <0.05 was considered to be statistically significant.

## Results

### ecSOD increases H_2_O_2_ level and promotes angiogenesis in ischemic hindlimbs, in a HBD-dependent manner

**Figure 1:**
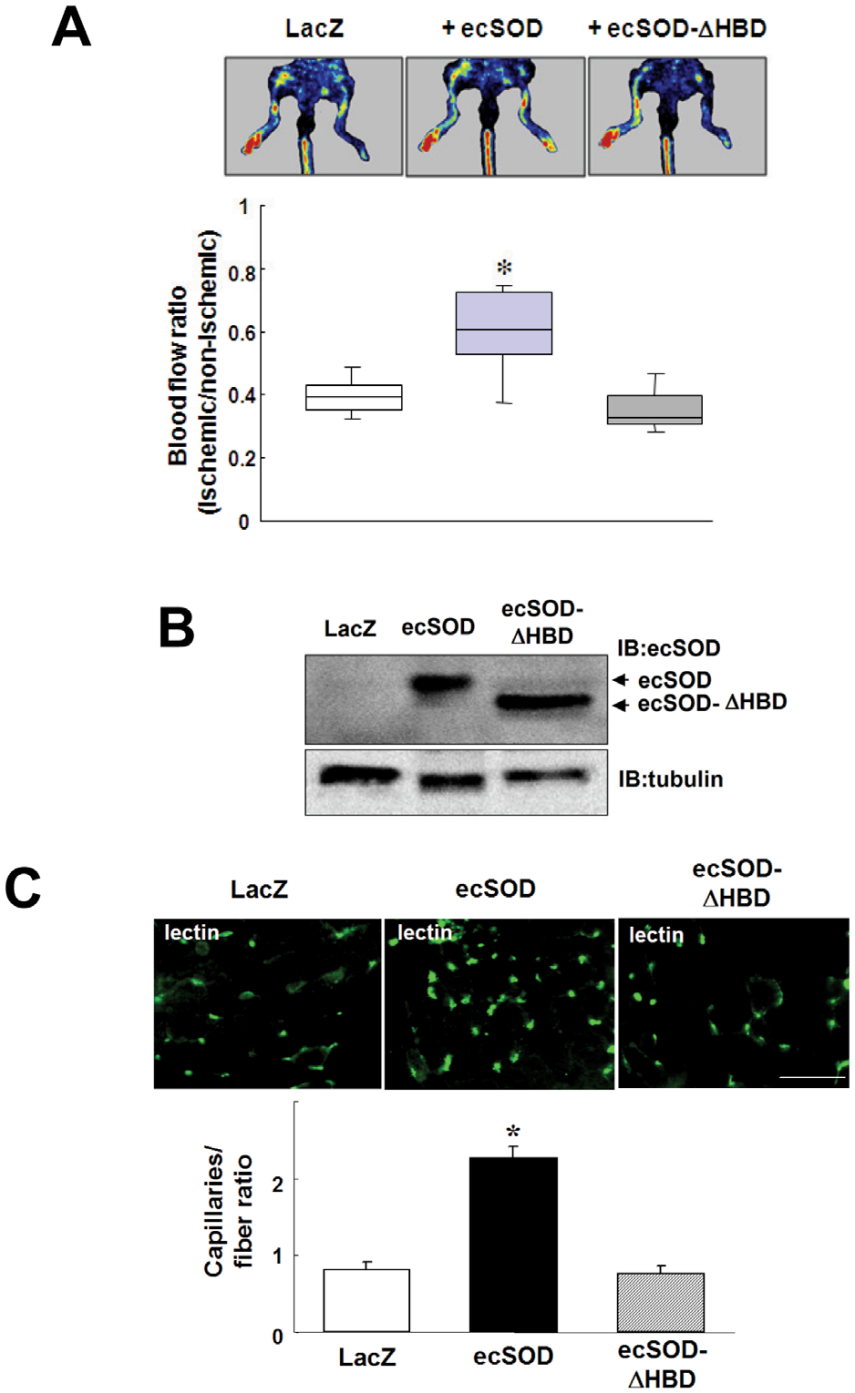
ecSOD gene transfer promotes blood flow recovery and angiogenesis in hindlimb ischemia model. **A.** C57BL/6J mice were subjected to unilateral hindlimb ischemic surgery and adenoviral injection (Ad.LacZ or Ad.ecSOD or Ad.ecSOD-ΔHBD, 1×10^9^ pfu) into adductor muscle was performed at immediately after surgery. Hindlimb blood flow recovery was measured by relative values of laser Doppler perfusion between ischemic and non-ischemic legs at day14 (n = 5–6). **B.** Representative Western blots of adductor muscle lysates obtained after adenoviral injection at day 3 probed by anti-human ecSOD or anti-α-tubulin antibodies. **C.** Mouse adductor muscle tissues were stained by *simplicifolia* lectin to detect capillaries at day7 after ischemia. Capillary density was quantitated as the number of capillaries per muscle fibers. (n = 4). Bar indicates 50 µm. *p<0.05 vs. Ad.LacZ.

**Figure 2:**
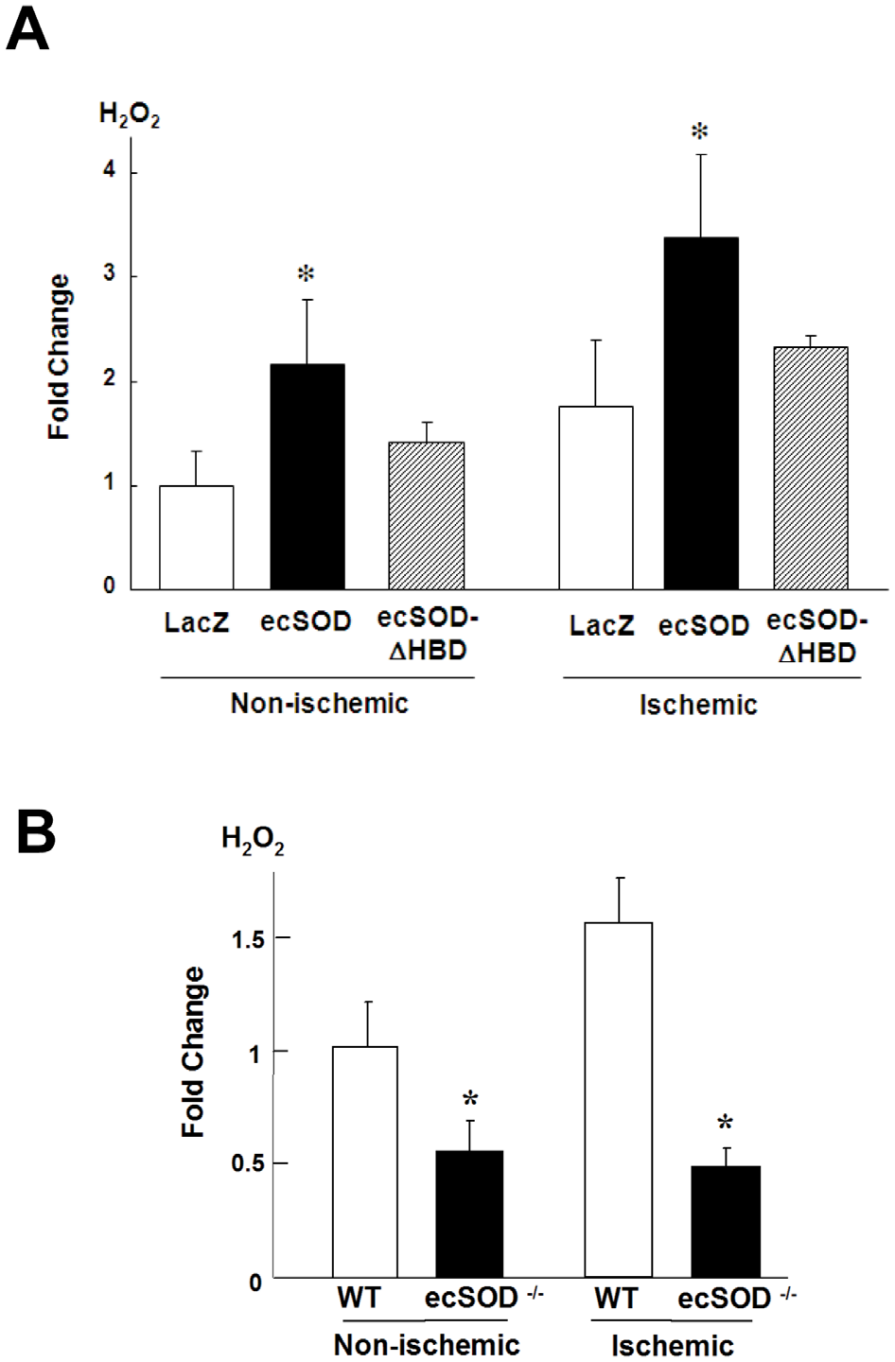
ecSOD increases H_2_O_2_ levels in non-ischemic and ischemic limbs in hindlimb ischemia model. H_2_O_2_ levels in non-ischemic and ischemic adductor muscles were measured by Amplex Red from WT mice after adenoviral injection (Ad.LacZ or Ad.ecSOD or Ad.ecSOD-ΔHBD, 1×10^9^ pfu) (**A**), or from WT and ecSOD^−/−^ mice (**B**) at day 3 (n = 4–6). The values were normalized by tissue weights and expressed as fold change over LacZ (A) or WT (B) of non-ischemic sites. *p<0.05 vs. LacZ (A) or WT (B).

To determine if ecSOD serves as a source of H_2_O_2_ and promotes angiogenesis *in vivo*, we injected Ad.ecSOD into adductor muscle immediately after hindlimb ischemia. Figure 1A shows that gene transfer of ecSOD, but not ecSOD-ΔHBD, improved limb blood flow recovery at day 14 after ischemia, as measured by laser Doppler blood flow analysis. Human specific ecSOD antibody confirmed the expression of both human ecSOD and ecSOD-ΔHBD proteins in adductor muscles at the similar extent (Fig. 1B). Figure 1C shows that Ad.ecSOD, but not Ad.ecSOD-ΔHBD, promoted ischemia-induced increase in capillary density, as detected by lectin staining. Figure 2A shows that Ad.ecSOD, but not Ad.ecSOD-ΔHBD, increased H_2_O_2_ levels in adductor muscle with or without hindlimb ischemia, as measured by Amplex Red, which predominantly detects extracellular H_2_O_2_. These suggest that ecSOD binding to tissue via HBD is required for its effects to increase H_2_O_2_ and promote angiogenesis in ischemic hindlimbs. We next examined whether endogenous ecSOD functions as a generator of H_2_O_2_ in ischemia hindlimb model. We previously demonstrated that ecSOD^−/−^ mice showed impaired ischemia-induced blood flow recovery and angiogenesis [17]. As shown in Figure 2B, hindlimb ischemia significantly increased H_2_O_2_ levels in adductor muscle of wild type (WT) mice, and that H_2_O_2_ levels in both non-ischemic and ischemic muscles were markedly reduced in ecSOD^−/−^ mice. These suggest that ecSOD is a predominant source of H_2_O_2_ in basal and after hindlimb ischemia, which may contribute to post-ischemic neovascularization.

### Extracellular H_2_O_2_ generated by ecSOD enhances VEGF-induced VEGFR2 autophosphorylation, in a HBD-dependent manner, in Ecs

**Figure 3:**
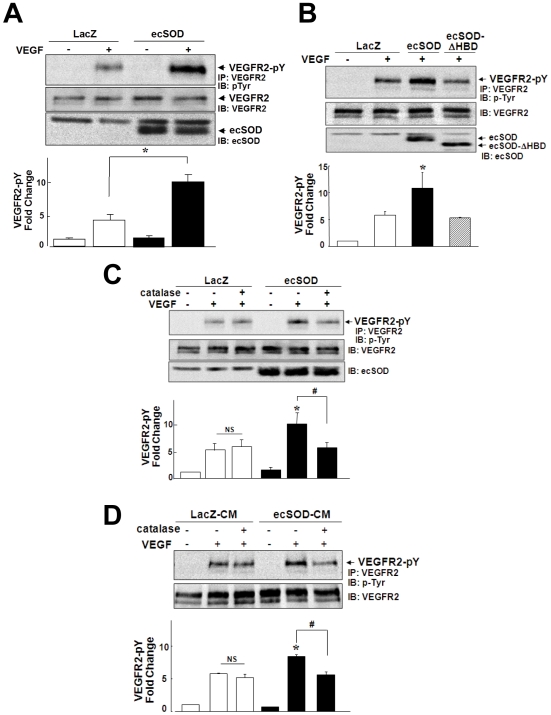
Extracellular H_2_O_2_ generated by ecSOD enhances VEGF-induced VEGFR2 autophosphorylation, in a HBD-dependent manner, in ECs. **A and B.** HUVECs were infected with Ad.ecSOD or Ad.LacZ (**A and B**) or Ad.ecSOD-ΔHBD (**B**), and stimulated with VEGF (20 ng/ml) for 5 min. Lysates were immunoprecipitated (IP) with anti-VEGFR2 antibody (Ab), followed by immunoblotted (IB) with anti-phospho-tyrosine (pTyr) Ab to measure VEGFR2-pY. The same lysates were IB with anti-VEGFR2 or ecSOD Abs (n = 3–4). **C.** HUVECs infected with Ad.LacZ or Ad.ecSOD were pretreated with catalase (500 U/ml) for 1 hr to scavenge extracellular H_2_O_2_, and then stimulated with VEGF (20 ng/ml) for 5 min. Lysates were used for measurement of VEGFR2-pY or total VEGFR2 or ecSOD expression (n = 4). **D.** HUVECs were incubated with conditioned media (CM) obtained from Ad.ecSOD or Ad.LacZ-infected HUVECs for 15 min, and stimulated with VEGF (20 ng/ml) for 5 min. Some cells were pretreated with catalase (500 U/ml) for 15 min to scavenge extracellular H_2_O_2_ before CM addition. Lysates were used for measurement of VEGFR2-pY or total VEGFR2 (n = 3). Bottom panel shows averaged data; expressed as fold change over basal (means ± S.E.). *p<0.05 vs. Ad.LacZ+VEGF. # p<0.05.

Since ecSOD anchoring to ECs surface via HBD is required for its EC protective function [16], we next examined the role of ecSOD-derived H_2_O_2_ in VEGF signaling in ECs. Figure 3A shows that infection of HUVECs with Ad.ecSOD significantly enhanced VEGF-induced VEGFR2 autophosphorylation without affecting basal phosphorylation. By contrast, Ad.ecSOD-ΔHBD had no effects on this response under the condition in which both ecSOD and ecSOD-ΔHBD were expressed in cell lysates to similar extent (Fig. 3B). We also verified the protein expression and activity of both ecSOD and ecSOD-ΔHBD in cultured media (Fig. S1). These suggest that newly synthesized ecSOD proteins pass through intracellular secretory pathway to the extracellular space, and that ecSOD bound to ECs surface via HBD, but not ecSOD inside the cells, is required for facilitating VEGF-induced VEGFR2-pY. Consistently, conditioned media of Ad.ecSOD-infected ECs also augmented VEGF-induced receptor phosphorylation (Fig. 3D). Of note, short-term pretreatment with the H_2_O_2_-detoxifying enzyme catalase that does not enter the cells prevented the effects induced by Ad.ecSOD (Fig. 3C) and conditioned media of Ad.ecSOD-infected ECs (Fig. 3D). By contrast, this exogenous catalase treatment had no effects on VEGF-induced VEGFR2 phosphorylation in LacZ-infected ECs. Either exogenous application of H_2_O_2_ (<500 µM) which is diffusible (Fig. S2), or NO donor DETA-NO (Fig. S3) had no effects on both basal and VEGF-induced VEGFR2-pY, while higher concentration of H_2_O_2_ (at 500 µM) only enhanced VEGF-induced this response (Fig. S2). We found that concentration of H_2_O_2_ in culture medium in Ad.ecSOD-infected ECs was at around 1 µM, as measured by Amplex Red. These suggest that extracellular H_2_O_2_ derived from ecSOD anchored to ECs surface via HBD is produced locally to promote VEGFR2 phosphorylation.

**Figure 4:**
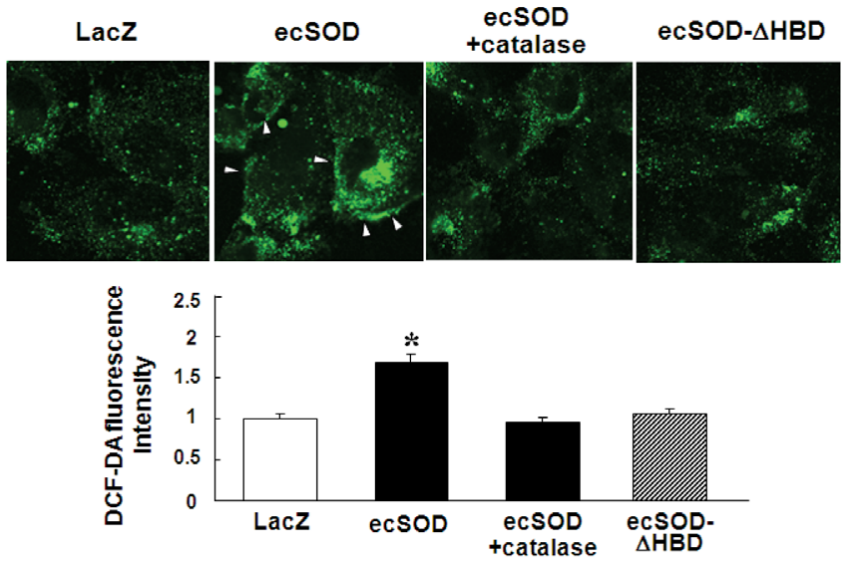
ecSOD increases H_2_O_2_ levels, in a HBD-dependent manner, in ECs. DCF fluorescence was measured by confocal microscopy in HUVECs infected with Ad.LacZ, Ad.ecSOD pretreated with or without catalase (500 U/ml, for 1 hr), or Ad.ecSOD-ΔHBD. Arrows indicate plasma membrane DCF staining. Lower panel shows the average of DCF fluorescence at 4 different fields (×63) (n = 4). * p<0.05 vs. Ad.LacZ.

We next examined whether ecSOD increases H_2_O_2_ levels in ECs using DCF-DA that detects intracellular peroxides including H_2_O_2_. Figure 4 shows that overexpression of ecSOD, but not ecSOD-ΔHBD, increased DCF fluorescence compared to Ad.LacZ-infected cells. Note that some of ecSOD-derived H_2_O_2_ signals accumulated at plasma membrane, and that short-term treatment of exogenous catalase inhibited ecSOD-induced DCF signal. We confirmed that pretreatment of ECs with polyethylene glycol (PEG)-catalase that enters the cells before loading DCF-DA abolished the fluorescence signals in basal state, as reported previously [38]. Taken together, these suggest that ecSOD binding to ECs via HBD is required to generate extracellular H_2_O_2_, which enters the cells to regulate VEGF signaling.

**Figure 5:**
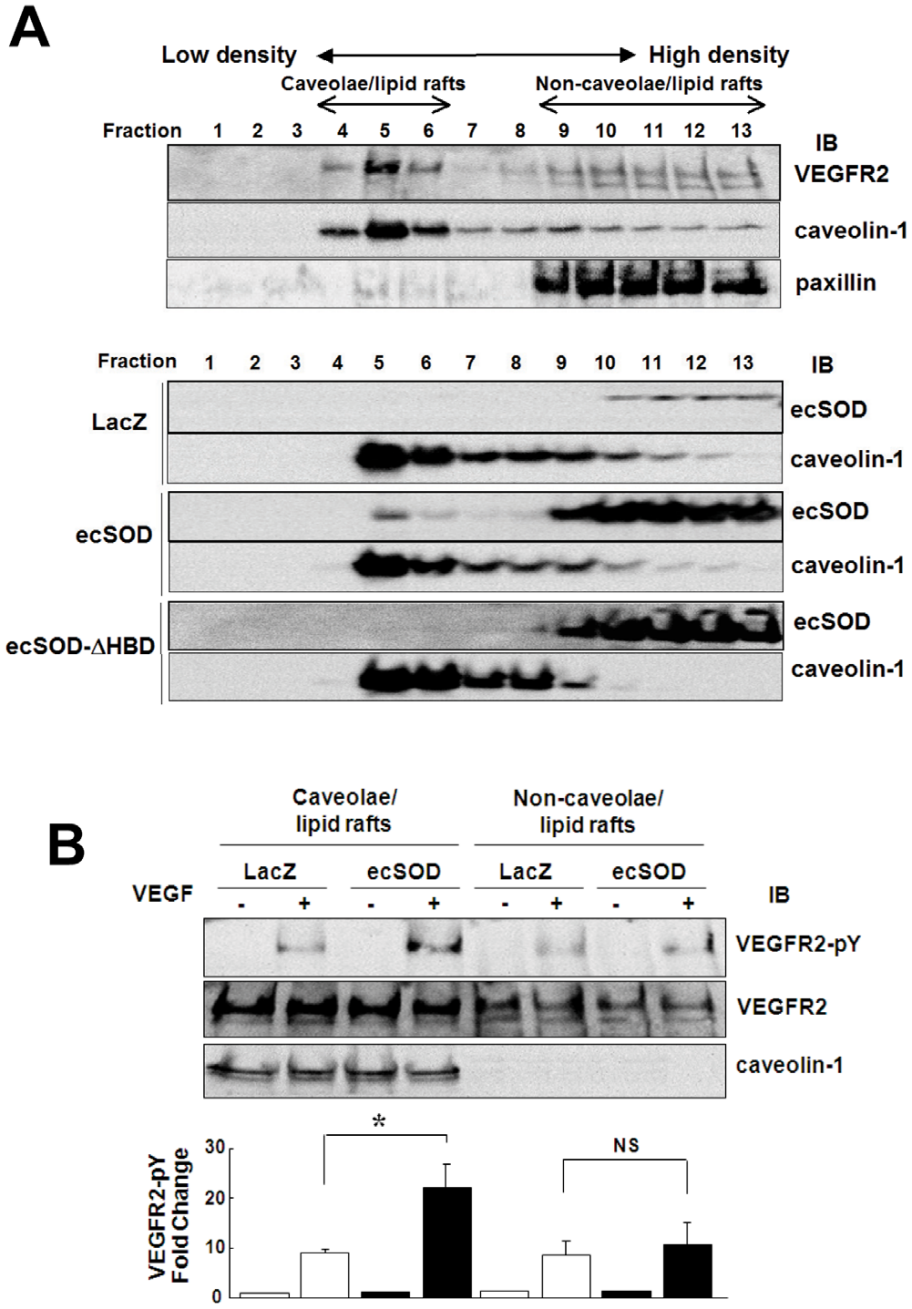
ecSOD localized in caveolae/lipid rafts via HBD enhances VEGF-induced VEGFR2 autophoshorylation in these microdomains. **A.** After sucrose gradient centrifugation to isolate caveolae/lipid rafts, equal volume of each fraction from top to bottom (total 13 fractions) was IB with anti-VEGFR2, caveolin-1, or paxillin Abs (upper panel). In lower panel, Ad.LacZ or Ad.ecSOD or Ad.ecSOD-ΔHBD-infected HUVECs were used for caveolae/lipid rafts isolation, and each fraction was IB with anti-ecSOD or caveolin-1 Abs. **B.** Ad.LacZ or Ad.ecSOD-infected HUVECs were stimulated with VEGF (20 ng/ml) for 5 min, and followed by caveolae/lipid rafts fractionation. Equal amounts of proteins from pooled Fraction 4–6 (caveolae/lipid rafts) and Fraction 9–13 (non-caveolae/lipid rafts) were IB with anti-VEGFR2-pY1175, total VEGFR2 or caveolin-1 Abs (n = 3). *p<0.05.

*ecSOD localized in caveolae/lipid rafts via HBD enhances VEGF-induced VEGFR2 autophoshorylation in these microdomains*. Since H_2_O_2_ is highly diffusible, and some core proteins of HSPGs and VEGFR2 are localized in caveolae/lipid rafts [19], [20], [28], we next examined whether ecSOD-induced regulation of VEGFR2 may occur in these microdomains. Sucrose gradient fractionation confirmed that VEGFR2 was localized in caveolin-1-enriched, low density lipid rafts fraction 4–6 (Fig. 5A). Intriguingly, ecSOD overexpression increased its localization in both caveolae/lipid rafts and non-caveolae/lipid rafts fractions (Fraction 9–13), while ecSOD-ΔHBD was found only in non-caveolae/lipid rafts (Fig. 5A). We verified the expression of both ecSOD and ecSOD-ΔHBD in total lysates (Fig. S4A). These indicate that HBD of ecSOD is required for its localization in lipid rafts, and that non-lipid rafts-localized ecSOD and ecSOD-ΔHBD may mainly represent their expression in the intracellular secretory pathway before secretion to the extracellular space. We also confirmed that endogenous ecSOD is found in caveolae/lipid rafts in mouse lung tissue which highly expresses ecSOD (Fig. S4B). Figure 5B shows that ecSOD overexpression selectively enhanced VEGF-induced VEGFR2 phosphorylation in caveolae/lipid rafts, but not non-caveolae/lipid rafts. Disruption of caveolae/lipid rafts by pretreatment with cholesterol-binding agent, methyl-β-cyclodextrin (MβCD) [19], [39], enhanced VEGF-induced VEGFR2 tyrosine phosphorylation, but completely inhibited ecSOD effects (Fig. S5). These suggest that ecSOD-induced augmentation of VEGFR2 activation is dependent on integrity of caveolae/lipid rafts.

**Figure 6:**
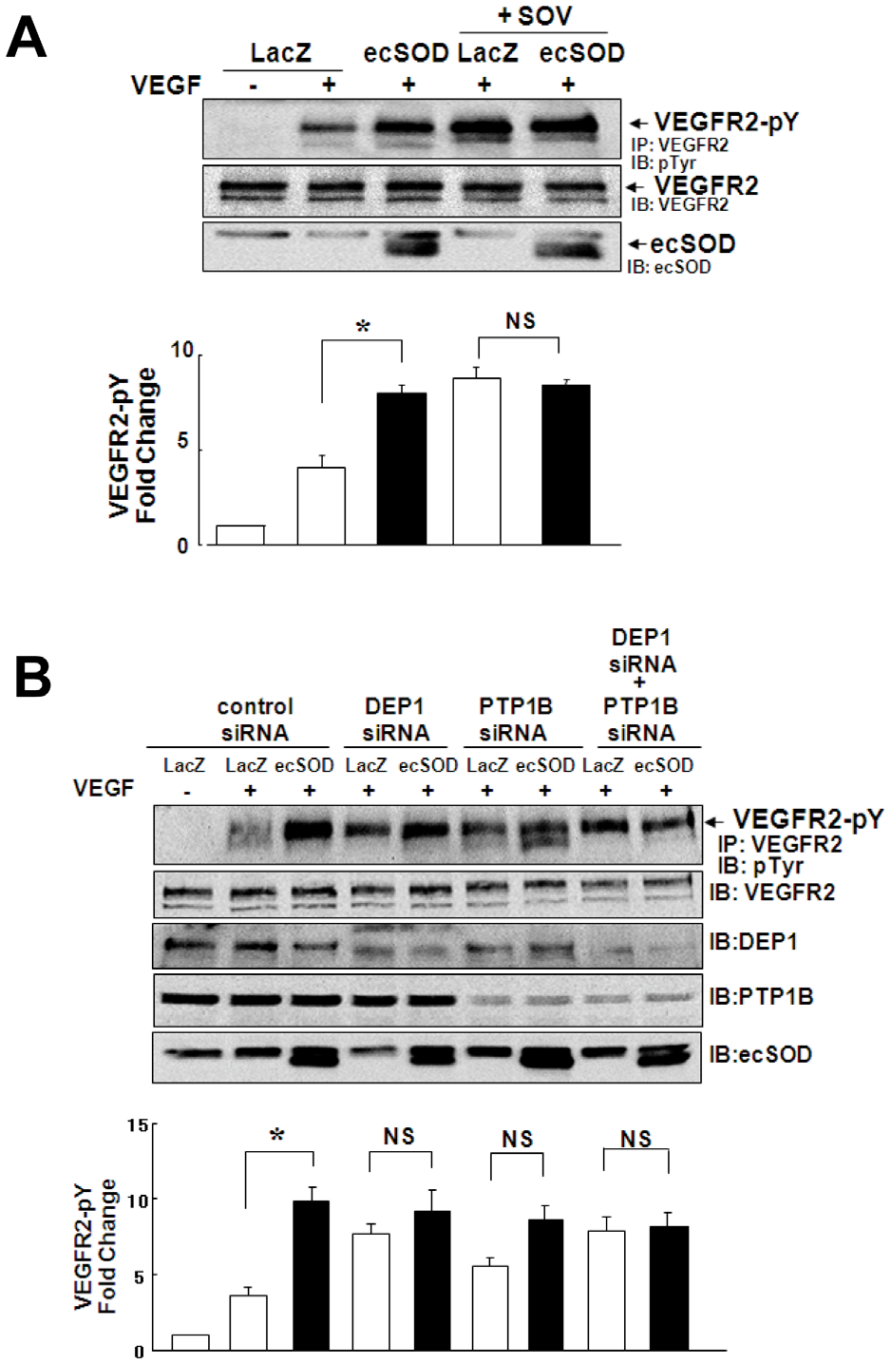
Inhibition of PTPs or knockdown of DEP1 and PTP1B prevents ecSOD-induced enhancement of VEGFR2 autophosphorylation. **A.** HUVECs infected with Ad.LacZ or Ad.ecSOD were pretreated with 0.3 mM sodium orthovanadate (SOV) for 30 min, and stimulated with VEGF (20 ng/ml) for 5 min. Lysates were used for measurement of VEGFR2-pY or total VEGFR2 or ecSOD expression (n = 3). **B.** HUVECs were transfected with DEP1 or/and PTP1B siRNA, and then infected Ad.LacZ or Ad.ecSOD. Cells were stimulated with VEGF (20 ng/ml) for 5 min and lysates were used for measurement of VEGFR2-pY and expression of proteins indicated (n = 4). * p<0.05.

*PTPs inhibition prevents ecSOD-induced enhancement of VEGF-induced VEGFR2 autophosphorylation*. To determine the mechanism by which ecSOD enhances VEGFR2 autophosphorylation, we examined whether ecSOD-derived H_2_O_2_ may inactivate PTPs such as DEP-1 and PTP1B, which negatively regulate VEGFR2 activation [24], [25]. Figure 6 shows that inhibition of PTPs by sodium orthovanadate (SOV) (Fig. 6A); or knockdown of either DEP-1 or PTP1B, or both proteins with siRNAs (Fig. 6B), significantly enhanced VEGF-induced VEGFR2-pY in LacZ infected cells. Either SOV or double knockdown of DEP1 and PTP1B almost completely prevented ecSOD-induced enhancement of VEGFR2 phosphorylation (Fig. 6A and 6B), while either DEP-1 siRNA or PTP1B siRNA alone partially but significantly blocked ecSOD effects. All these treatments had no effects on basal VEGFR2 phosphorylation (data not shown). These results suggest that ecSOD-induced enhancement of VEGF-induced VEGFR2-pY is mediated at least through inhibition of DEP-1 and/or PTP1B.

### ecSOD induces oxidative inactivation of DEP1 and PTP1B localized in caveolae/lipid rafts

**Figure 7:**
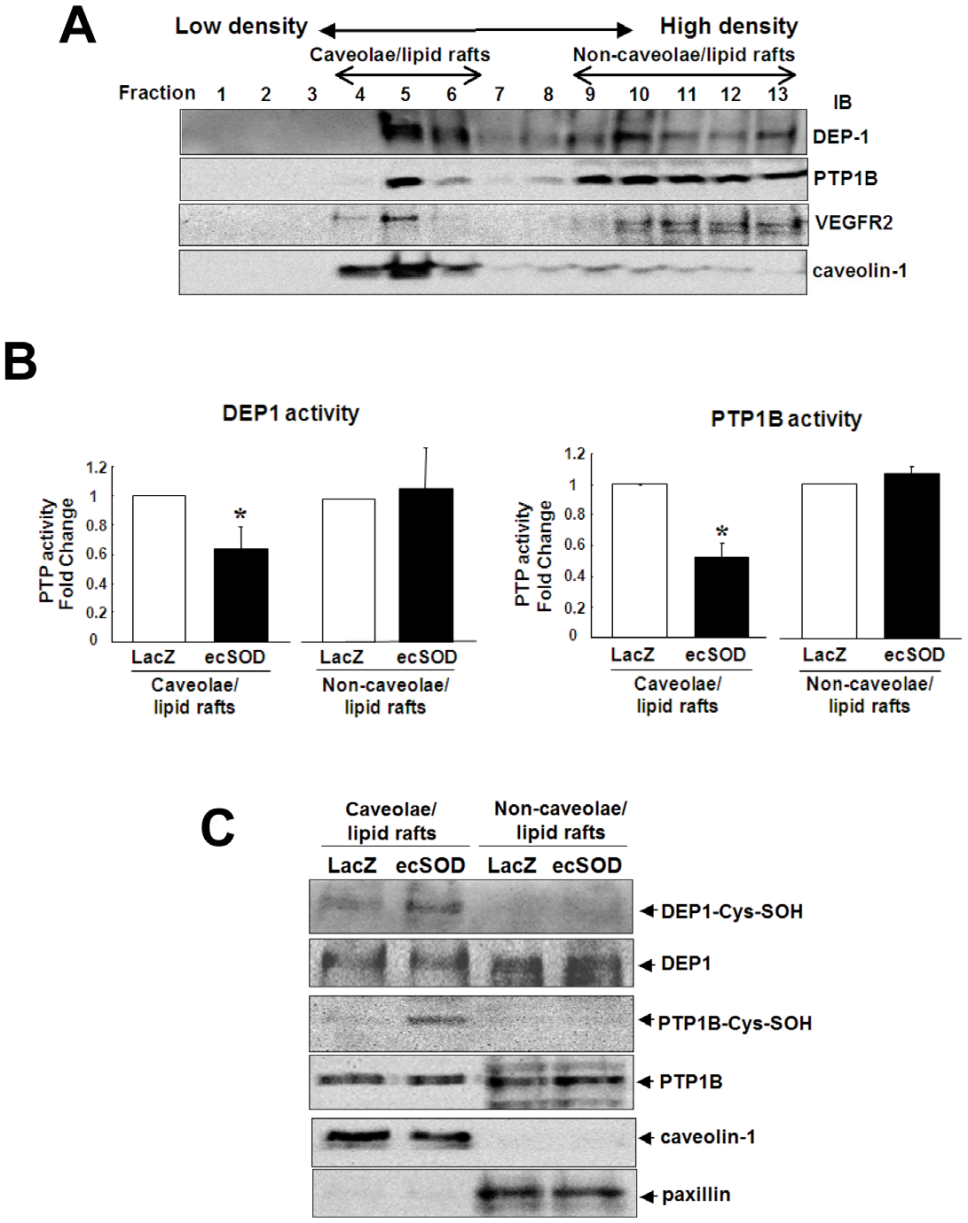
ecSOD induces inactivation and oxidation of DEP-1 and PTP1B localized in caveolae/lipid rafts. **A.** After sucrose gradient centrifugation, equal volumes of each fraction from top to bottom (total 13 fractions) were IB with anti-DEP-1, PTP1B, VEGFR2 or caveolin-1 Abs. **B.** DEP-1 and PTP1B activities in pooled Fraction 4–6 (caveolae/lipid rafts) and Fraction 9–13 (non-caveolae/lipid rafts) in Ad.LacZ and Ad.ecSOD-infected HUVECs were measured using pNPP as a substrate after IP with anti-DEP-1, PTP1B Abs. The values were expressed as a ratio to Ad.LacZ infected PTP activity (defined as 1.0) in each fraction (n = 3) *p<0.05. **C.** Ad.LacZ or Ad.ecSOD-infected HUVECs were extracted in the presence of biotin-labeled Cys-SOH trapping reagent DCP-Bio1. After sucrose gradient centrifugation, pooled Fraction 4–6 and Fraction 9–13 were affinity captured with streptavidin beads to purify the Cys-SOH formed protein, followed by IB with anti-DEP-1 or PTP1B Abs.

Since PTPs are inactivated by ROS via reactive Cys oxidation [4], [5], we next examined whether DEP1 and PTP1B are localized in caveolin-enriched lipid rafts, and oxidized by ecSOD. Figure 7A shows that both DEP1 and PTP1B are found in both caveolae/lipid rafts and non-caveolae/lipid rafts fractions, and that ecSOD overexpression decreased their PTP activity in caveolae/lipid rafts, but not non-caveolae/lipid rafts (Fig. 7B). Furthermore, newly-developed Cys-SOH trapping reagent [36] revealed that Ad.ecSOD increased Cys-OH formation of DEP1 and PTP1B in lipid rafts fraction. These suggest that extracellular H_2_O_2_ generated by ecSOD induces oxidative inactivation of DEP1/PTP1B in caveolae/lipid rafts, thereby promoting VEGF-induced VEGFR2 autophosphorylation in these specialized microdomains.

### ecSOD promotes VEGF-induced EC migration

**Figure 8:**
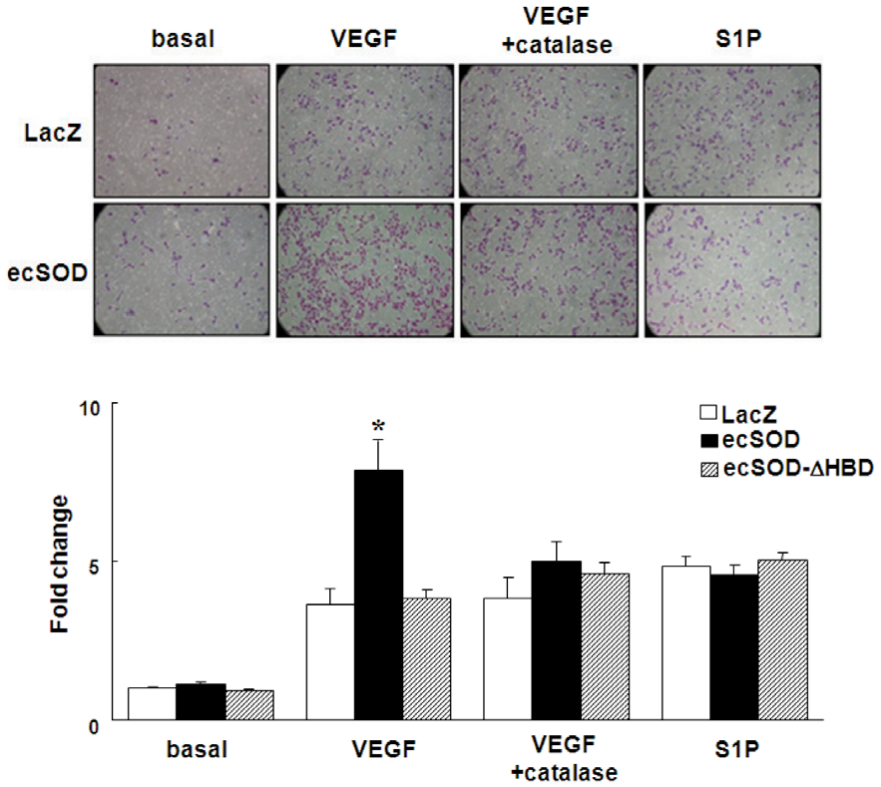
ecSOD promotes VEGF-induced EC migration in a HBD-dependent manner. HUVECs infected with Ad.LacZ or Ad.ecSOD or Ad.ecSOD-ΔHBD were stimulated with 20 ng/ml VEGF or 10 µmol/L sphingosine-1-phosphate (S1P) for 6 hours, and cell migration was measured by the modified Boyden chamber method. Some cells were pretreated with 500 U/ml catalase for 15 min and performed migration assay in the presence of catalase. Bar graph represents averaged data, expressed as cell number counted per 10 fields (x200) and fold change over that in unstimulated cells (control). * p<0.05 vs. Ad.LacZ+VEGF.

We next examined the functional consequence of enhancement of VEGFR2 activation by ecSOD-derived extracellular H_2_O_2_ in VEGF-induced EC migration and proliferation. Figure 8 using modified Boyden chamber assay shows that ecSOD, but not ecSOD-ΔHBD, significantly enhanced VEGF-induced migration without affecting sphingosine-1-phosphate (S1P)-induced response. Thus, ecSOD-induced effect is specific for VEGFR2 signaling. Importantly, ecSOD-induced enhancement of VEGF-induced EC migration was prevented by catalase, supporting the role of ecSOD-derived H_2_O_2_. VEGF-induced EC proliferation was also augmented by Ad.ecSOD (Fig. S6). These effects of ecSOD were associated with an enhancement of VEGFR2 downstream signaling such as PLCγ and p38MAPK phosphorylation (Fig. S7).

## Discussion

The present study provides novel evidence that ecSOD functions as a generator of extracellular H_2_O_2_ in specific subcellular compartments to promote VEGF signaling linked to angiogenesis. Here we show that: 1) gene transfer of ecSOD, but not ecSOD-ΔHBD, increases H_2_O_2_ levels in adductor muscle, and promotes angiogenesis in response to hindlimb ischemia; 2) H_2_O_2_ levels in both non-ischemic and ischemic hindlimbs are markedly reduced in ecSOD^−/−^ mice; 3) *In vitro*, overexpression of ecSOD, but not ecSOD-ΔHBD, in cultured medium in ECs enhances VEGF-induced VEGFR2-pY through generation of extracellular H_2_O_2_; 4) HBD of ecSOD is required for localization of ecSOD at plasma membrane caveolin-enriched lipid rafts where VEGFR2 and PTP1B/DEP-1 are found; 5) endogenous ecSOD is also found in caveolae/lipid rafts in tissues enriched with ecSOD; 6) VEGF-induced VEGFR2-pY in caveolae/lipid rafts, but not non-lipid rafts, is selectively enhanced by ecSOD, which is at least due to oxidative inactivation of PTP1B and DEP1 in caveolae/lipid rafts; 7) ecSOD-derived H_2_O_2_ promotes VEGF-induced EC migration in a HBD-dependent manner.

Exogenous H_2_O_2_ can increase angiogenic gene expression and promote pro-angiogenesis responses in ECs [8], [13]. However, since H_2_O_2_ is diffusible and short-lived, its application for therapeutic neovascularization *in vivo* is difficult and not efficient. ecSOD is the enzyme that catalyzes dismutation of O_2_^−^ to produce H_2_O_2_ in the extracellular space by anchoring to ECs surface or extracellular matrix through HBD [14]. We previously reported that ecSOD expression is increased in response to hindlimb ischemia, and that post-ischemic revascularization is impaired in ecSOD^−/−^ mice [17]. However, a role of ecSOD-derived H_2_O_2_ in VEGF signaling and ischemia-induced angiogenesis was virtually unexplored. Here we show that gene transfer of Ad.ecSOD, but not Ad.ecSOD-ΔHBD, increases H_2_O_2_ production in adductor muscles, as measured by Amplex Red assay, which predominantly detects extracellular H_2_O_2_, as well as promotes blood flow recovery and capillary formation in response to hindlimb ischemia. Furthermore, ecSOD^−/−^ mice show significant reduction of H_2_O_2_ levels in both non-ischemic and ischemic hindlimbs. These results strongly suggest that ecSOD bound to tissue via HBD plays an important to role as a generator of extracellular H_2_O_2_ to promote angiogenesis *in vivo*. To determine the underlying mechanisms, we examined the effects of ecSOD-derived H_2_O_2_ on VEGF signaling in ECs. The present study demonstrates for the first time that overexpression of ecSOD, but not ecSOD-ΔHBD, in ECs or its conditioned media enhances VEGF-induced VEGFR2 autophosphorylation. Moreover, these ecSOD-induced effects on VEGFR2, but not VEGF-induced VEGFR2 autophosphorylation, are inhibited by short-term pretreatment with catalase that scavenges extracellular H_2_O_2_. Thus, these findings indicate that extracellular H_2_O_2_ derived from ecSOD promotes VEGF-induced VEGFR2-pY in ECs in a HBD-dependent manner.

In this study, we found that H_2_O_2_ concentration in culture media of Ad.ecSOD-infected ECs is around 1 µM, while exogenous H_2_O_2_ requires at least 500 µM to enhance VEGF-induced receptor phosphorylation. These results support the possibility that ecSOD binding to ECs surface via HBD may provide the microenvironment in which extracellular H_2_O_2_ generated by ecSOD is more compartmentalized than exogenously-applied H_2_O_2_. Of note, either high concentration of exogenous H_2_O_2_ or Ad.ecSOD has no effects on basal VEGFR2-pY. These suggest that ligand-induced pre-assembly of VEGFR2 containing signaling complexes and/or their specific localization might be required for promoting effect of extracellular H_2_O_2_ derived from ECs-bound ecSOD on VEGFR2-pY. It has been shown that VEGF-induced VEGFR2 autophosphorylation is regulated by “intracellular” H_2_O_2_ derived from Nox2-based NADPH oxidase in ECs [7], [8]. NADPH oxidase-dependent O_2_^−^ production occurs both intracellularly and extracellularly [12], [40]. Thus, NADPH oxidase-derived O_2_^−^ produced extracellularly may be rapidly dismutated by ecSOD to generate H_2_O_2_ in close proximity to the VEGFR2 to facilitate its phosphorylation efficiently. Of note, classical role of ecSOD is to scavenge O_2_^−^ to increase NO bioactivity; however, NO donor has no effect on VEGF-induced phosphorylation of the VEGFR2. Thus, it is H_2_O_2_ rather than NO, which mediates ecSOD-induced augmentation of VEGFR2 activation in ECs.

ecSOD binds to cell surface HSPGs via HBD, and some cell surface core proteins of HSPGs are localized in caveolae/lipid rafts in ECs [28], [41]. We thus examined whether ecSOD-induced modulation of VEGFR2 might occur in these specialized microdomains. Sucrose gradient fractionation reveals that ecSOD is localized in both caveolae/lipid rafts and non-caveolae/lipid rafts fractions in Ad.ecSOD-infected ECs, while ecSOD-ΔHBD is found only in non-caveolae/lipid rafts fraction. Of note, endogenous ecSOD protein is also found in caveolae/lipid rafts in lung tissue in which ecSOD is abundantly expressed. We show that VEGF-induced VEGFR2-pY in caveolae/lipid rafts, but not in non-caveolae/lipid rafts, is enhanced by ecSOD. Disruption of caveolae/lipid rafts by cholesterol-binding reagent increases VEGF-induced VEGFR2 autophosphorylation, but prevents ecSOD-induced effect. Mechanism by which cholesterol depletion increases VEGF-induced phosphorylation of VEGFR2 in ECs seems to be due to dissociation of VEGFR2 from caveolin [19]. Thus, these results suggest that ecSOD localization at caveolin-enriched lipid rafts via HBD is required for ecSOD-induced enhancement of ligand-induced VEGFR2 phosphorylation in these specific plasma membrane compartments.

Reversible oxidative inactivation of PTPs by ROS [42], [43], [44] and their specific localization are important for ROS to increase tyrosine phosphorylation signaling events [4], [5]. The initial product of Cys oxidation is Cys-SOH, a key intermediate involved in redox signaling [45]. The present study shows that inhibition of PTPs or knockdown of DEP-1 and/or PTP1B increases VEGF-induced VEGFR2-pY, which prevents ecSOD-induced effect on VEGFR2. These suggest that both DEP1 and PTP1B function as a negative regulator for VEGFR2-pY, as reported previously [24], [25], and that ecSOD-derived H_2_O_2_ inhibits their PTPs activity to promote VEGFR2 phosphorylation. Intriguingly, we found that both DEP1 and PTP1B are localized in both caveolae/lipid rafts and non-lipid rafts in ECs. Moreover, newly-developed cell permeable Cys-SOH trapping probe [36], [37] reveals that ecSOD increases Cys-SOH formation of DEP-1 and PTP1B as well as decreases their PTP activity in caveolin-enriched lipid rafts, but not in non-lipid rafts. NADPH oxidase is localized in lipid rafts to generate O_2_^−^ in ECs [21]. These suggest that extracellular H_2_O_2_ generated by ecSOD locally oxidizes and inactivates DEP-1 and/or PTP1B in caveolae/lipid rafts where NADPH oxidase and VEGFR2 are found, which in turn promotes VEGF-induced VEGFR2 phosphorylation in these specific microdomains. Other possible PTPs that are regulated by ecSOD cannot be ruled out in the current study.

Functionally, ecSOD, but not ecSOD-ΔHBD, promotes VEGF-induced EC migration *in vitro*, which is prevented by exogenous application of catalase. This is consistent with ecSOD-induced augmentation of ischemia-induced angiogenesis *in vivo*. Of note, S1P-induced migration was not affected by Ad.ecSOD, supporting our conclusion that localizing ecSOD, VEGFR2, and DEP-1/PTP1B in lipid rafts as important mechanism by which ecSOD-derived H_2_O_2_ enhances VEGFR2 signaling lined to angiogenic responses. We previously reported that ecSOD functions to preserve NO bioactivity by scavenging O_2_^−^ in the ischemic tissues, thereby promoting angiogenesis [17]. Similarly, HBD-dependent protective endothelial function of ecSOD via decreasing extracellular O_2_^−^ has been reported in animal model with hypertension [16]. The R213G polymorphism in the ecSOD gene, which reduces binding to endothelium surface and increases serum ecSOD levels, is associated with increased risk of cardiovascular diseases [46]. The present study uncovers a novel mechanism by which ecSOD promotes endothelial functions such as EC migration and proliferation by generating extracellular H_2_O_2_ at the specific membrane compartment, and thus facilitating VEGF signaling linked to angiogenesis. In contrast, ecSOD overexpression inhibits, instead of increase, tumor angiogenesis and tumor invasion [47], [48]. In pro-oxidant pathological conditions such as atherosclerosis and hypertension, ecSOD seems to be inactivated by H_2_O_2_ derived from ecSOD due to its peroxidase activity [49], [50]. Thus, ecSOD gene transfer effect on angiogenesis *in vivo* seems to be varied with cell types and context specific [17], [47], [48], [51].

**Figure 9:**
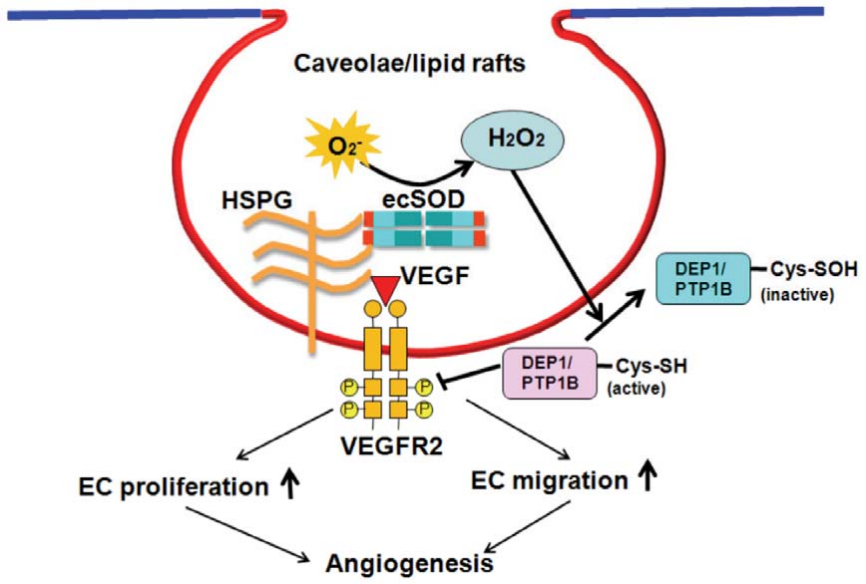
Proposed model for role of ecSOD-derived H_2_O_2_ in VEGFR2 signaling linked to angiogenesis. Extracellular H_2_O_2_ generated by ecSOD localized at caveolae/lipid rafts via HBD induces oxidative inactivation of DEP1 and PTP1B in these microdomains, thereby promoting VEGF-induced VEGFR2 phosphorylation, which may contribute to EC migration and proliferation *in vitro* as well as angiogenesis *in vivo*.

In summary, extracellular H_2_O_2_ generated by ecSOD localized at caveolin-enriched lipid rafts via HBD efficiently facilitates VEGFR2 signaling via oxidative inactivation of DEP-1/PTP1B in these microdomains, which may contribute to promoting postnatal angiogenesis (Fig. 9). Our previous and present studies may uncover novel mechanism whereby increased ecSOD expression in ischemic tissues promotes reparative neovascularization *in vivo*. It is likely that ecSOD may serve as a potent generator of extracellular H_2_O_2_ in the plasma membrane specific compartments to promote angiogenesis growth factor signaling. The present findings also imply that ecSOD gene transfer may represent an important therapeutic approach for treatment of angiogenesis-dependent diseases including ischemic heart and limb diseases.

## Supporting Information

Figure S1 ecSOD and ecSOD-ΔHBD protein expression and activity in culture medium in adenovirus infected HUVECs. Conditioned media obtained from HUVECs infected with Ad.LacZ or Ad.ecSOD or Ad.ecSOD-ΔHBD was used for Western analysis with anti-human ecSOD antibody (A) or measurement of ecSOD activity (B).
(0.03 MB PDF)

Figure S2 Exogenous H_2_O_2_ at physiological concentration cannot enhance VEGF-induced VEGFR2 autophosphorylation. HUVECs were pretreated with indicated concentration of H_2_O_2_ for 15 min, and stimulated with VEGF (20 ng/ml) for 5 min. Lysates were immunoprecipitated (IP) with anti-VEGFR2 Ab and followed by immunoblotted (IB) with anti-pTyr Ab for measurement of VEGFR2-pY (n = 3).
(0.05 MB PDF)

Figure S3 Exogenous application of NO donor has no effect on VEGF-induced VEGFR2 autophosphorylation. HUVECs were pretreated with indicated concentration of No donor, diethylenetetraamine-NONOate (DETA-NO) for 30 min, and stimulated with VEGF (20 ng/ml) for 5 min. Lysates were used for measurement of VEGFR2-pY.
(0.05 MB PDF)

Figure S4 Endogenous ecSOD is localized in caveolae/lipid rafts in mouse lung in which ecSOD is highly expressed. A. Total lysates from HUVECs infected Ad.LacZ or Ad.ecSOD or Ad.ecSOD-ΔHBD for caveolae isolation were IB with anti-ecSOD to confirm the expression of ecSOD and ecSOD-ΔHBD. B. Mouse lung (400 mg) was fractionated to isolate caveolae/lipid rafts and IB with anti-mouse ecSOD or caveolin-1 antibodies.
(0.05 MB PDF)

Figure S5 Intact caveolae/lipid rafts are required for ecSOD-induced enhancement of VEGFR2 autophosphorylation. HUVECs were pretreated with or without 10 mM methyl-β-cyclodextrin (MβCD) for 1 hr, and stimulated with VEGF (20 ng/ml) for 5 min. Lysates were used for measurement of VEGFR2-pY or total VEGFR2 or ecSOD expression (n = 3). * p<0.05.
(0.09 MB PDF)

Figure S6 ecSOD promotes VEGF-induced EC proliferation. Ad.LacZ or Ad.ecSOD-infected HUVECs were cultured in 0.5% FBS containing medium with or without VEGF (20 ng/ml) for 48 hours, and cell number was counted with a hemocytometer (n = 8). * p<0.05.
(0.01 MB PDF)

Figure S7 ecSOD enhances VEGFR2 downstream signaling in HUVECs. Cell lysates from Ad.LacZ and Ad.ecSOD infected HUVECs with or without VEGF stimulation (20 ng/ml, 5 min) were IB with anti-p-PLCγ or PLCγ (A) or p-p38MAPK or p38MAPK (B) antibodies (n = 3). *p<0.05
(0.08 MB PDF)
