# Supplementary figures and images for: Extracellular SOD-Derived H2O2 Promotes VEGF Signaling in Caveolae/Lipid Rafts and Post-Ischemic Angiogenesis in Mice
Source: PLoS One. 2010 Apr 21;5(4):e10189. doi: 10.1371/journal.pone.0010189 (PMC2858087; doi:10.1371/journal.pone.0010189)

# Supplemental Figure S1

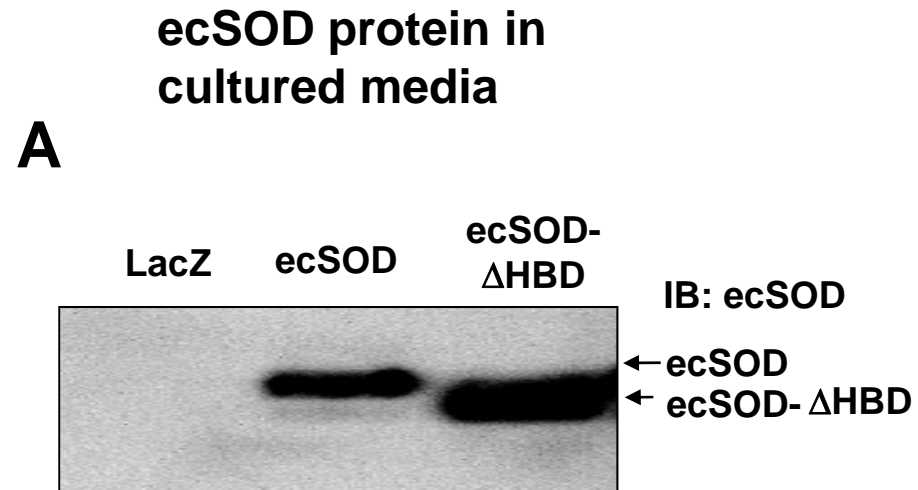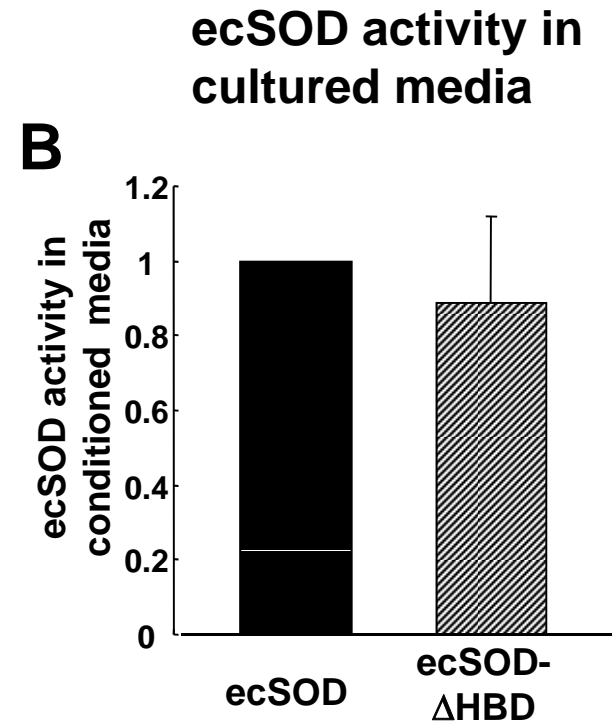

Supplement: Figure S1 — ecSOD and ecSOD-ΔHBD protein expression and activity in culture medium in adenovirus infected HUVECs. Conditioned media obtained from HUVECs infected with Ad.LacZ or Ad.ecSOD or Ad.ecSOD-ΔHBD was used for Western analysis with anti-human ecSOD antibody (A) or measurement of ecSOD activity (B). (0.03 MB PDF) [file pone.0010189.s001.pdf]

## Supplemental Figure S2

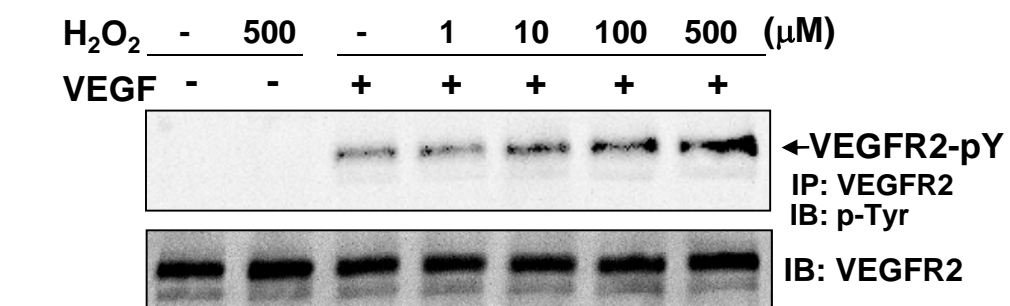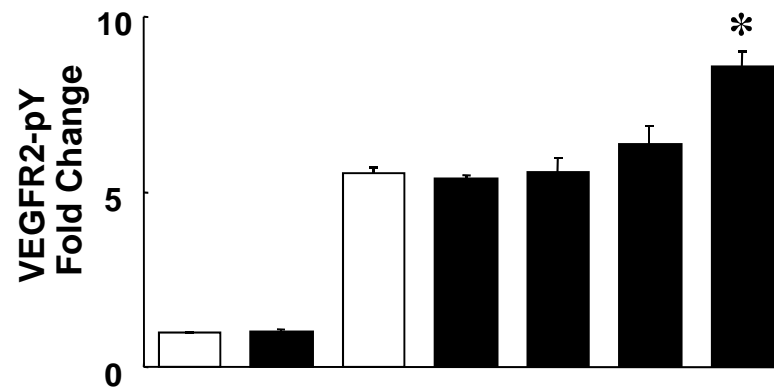

Supplement: Figure S2 — Exogenous H2O2 at physiological concentration cannot enhance VEGF-induced VEGFR2 autophosphorylation. HUVECs were pretreated with indicated concentration of H2O2 for 15 min, and stimulated with VEGF (20 ng/ml) for 5 min. Lysates were immunoprecipitated (IP) with anti-VEGFR2 Ab and followed by immunoblotted (IB) with anti-pTyr Ab for measurement of VEGFR2-pY (n = 3). (0.05 MB PDF) [file pone.0010189.s002.pdf]

## Supplemental Figure S3

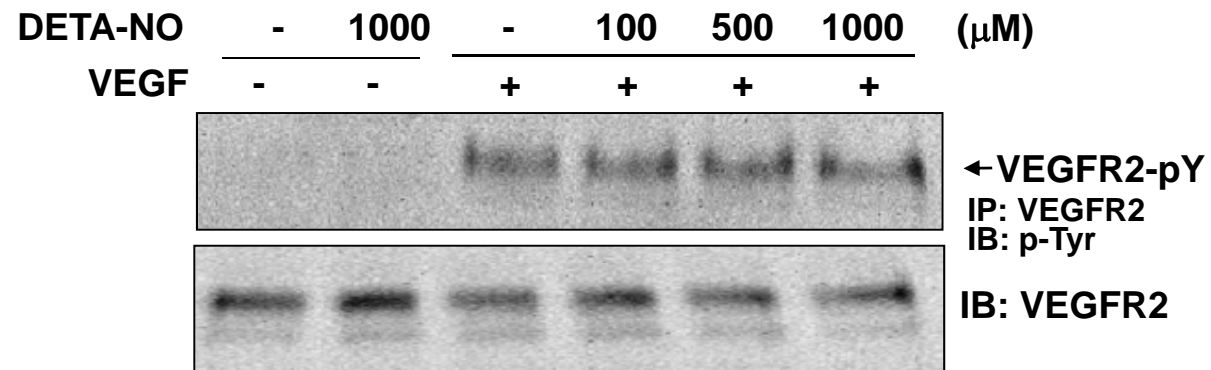

Supplement: Figure S3 — Exogenous application of NO donor has no effect on VEGF-induced VEGFR2 autophosphorylation. HUVECs were pretreated with indicated concentration of No donor, diethylenetetraamine-NONOate (DETA-NO) for 30 min, and stimulated with VEGF (20 ng/ml) for 5 min. Lysates were used for measurement of VEGFR2-pY. (0.05 MB PDF) [file pone.0010189.s003.pdf]

# Supplemental Figure S4

**A**

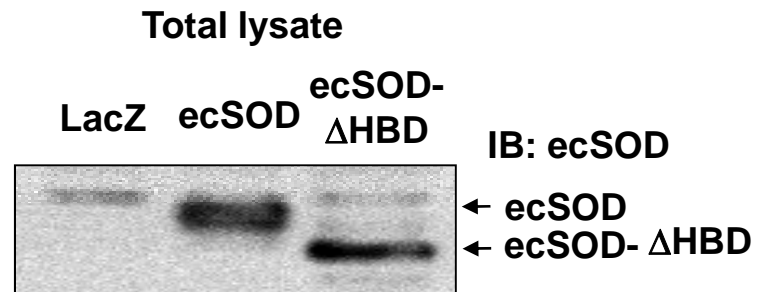

**B**

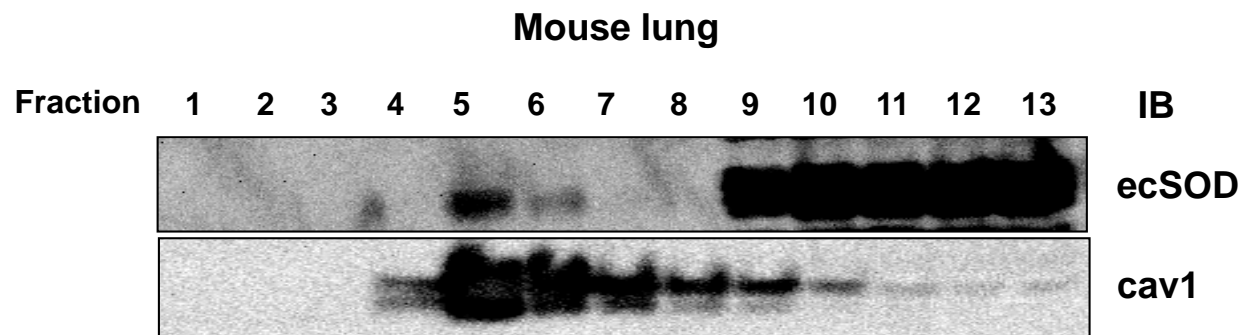

Supplement: Figure S4 — Endogenous ecSOD is localized in caveolae/lipid rafts in mouse lung in which ecSOD is highly expressed. A. Total lysates from HUVECs infected Ad.LacZ or Ad.ecSOD or Ad.ecSOD-ΔHBD for caveolae isolation were IB with anti-ecSOD to confirm the expression of ecSOD and ecSOD-ΔHBD. B. Mouse lung (400 mg) was fractionated to isolate caveolae/lipid rafts and IB with anti-mouse ecSOD or caveolin-1 antibodies. (0.05 MB PDF) [file pone.0010189.s004.pdf]

## Supplemental Figure S5

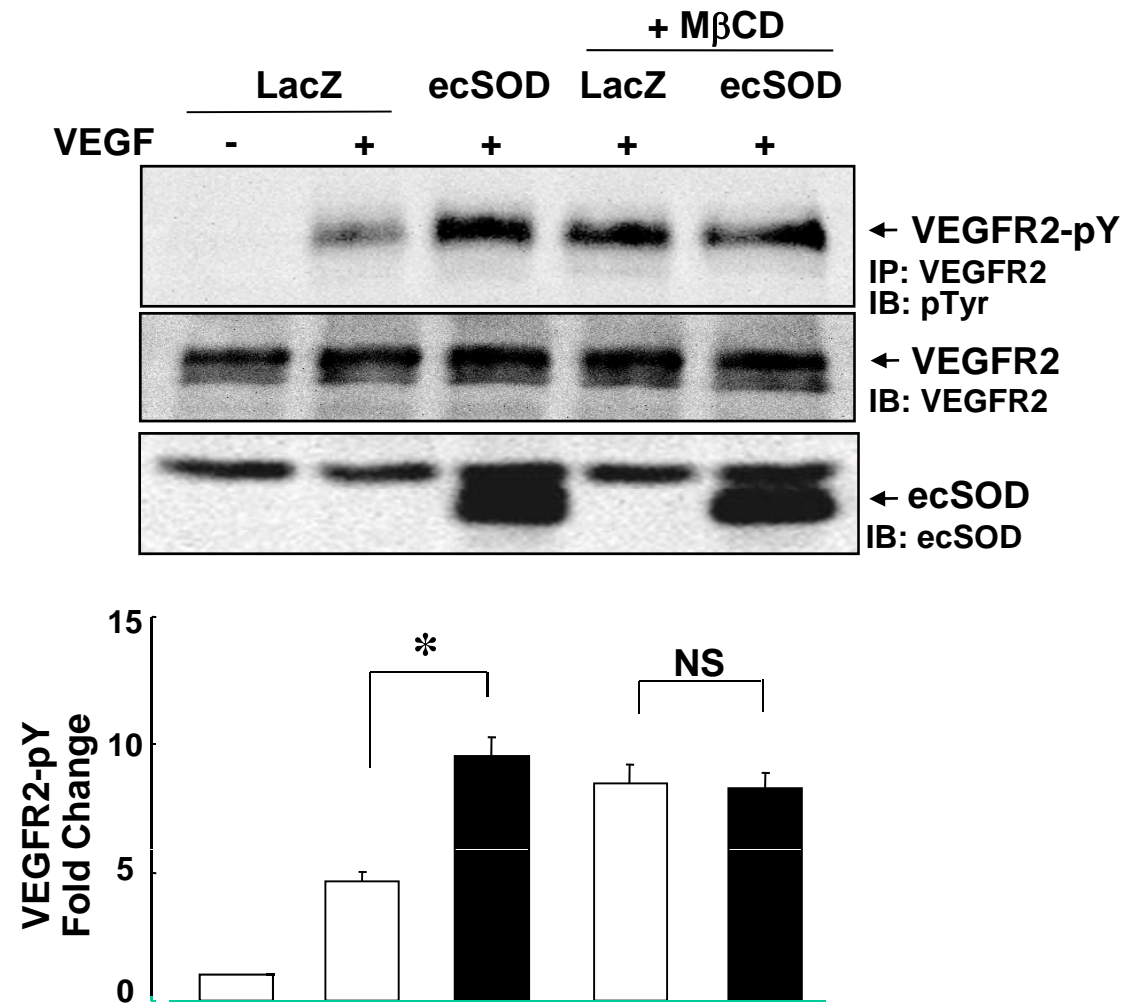

Supplement: Figure S5 — Intact caveolae/lipid rafts are required for ecSOD-induced enhancement of VEGFR2 autophosphorylation. HUVECs were pretreated with or without 10 mM methyl-β-cyclodextrin (MβCD) for 1 hr, and stimulated with VEGF (20 ng/ml) for 5 min. Lysates were used for measurement of VEGFR2-pY or total VEGFR2 or ecSOD expression (n = 3). * p<0.05. (0.09 MB PDF) [file pone.0010189.s005.pdf]

## Supplemental Figure S6

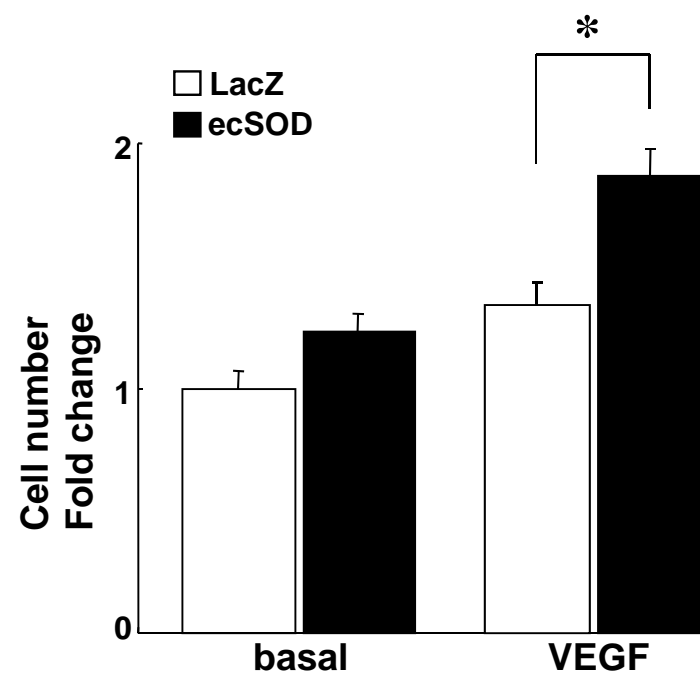

Supplement: Figure S6 — ecSOD promotes VEGF-induced EC proliferation. Ad.LacZ or Ad.ecSOD-infected HUVECs were cultured in 0.5% FBS containing medium with or without VEGF (20 ng/ml) for 48 hours, and cell number was counted with a hemocytometer (n = 8). * p<0.05. (0.01 MB PDF) [file pone.0010189.s006.pdf]

# Supplemental Figure S7

**A**

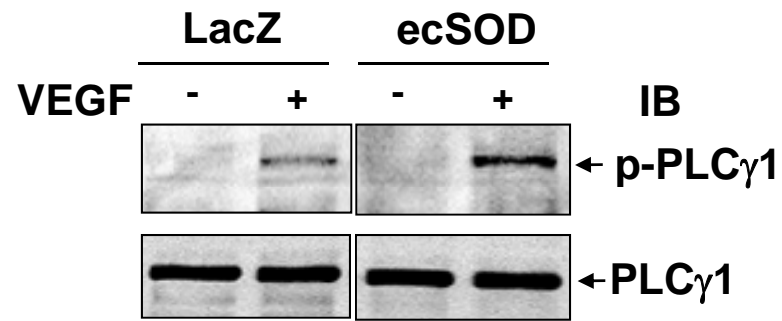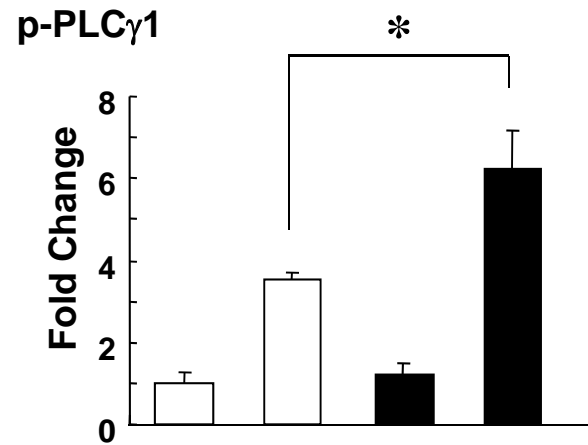

**B**

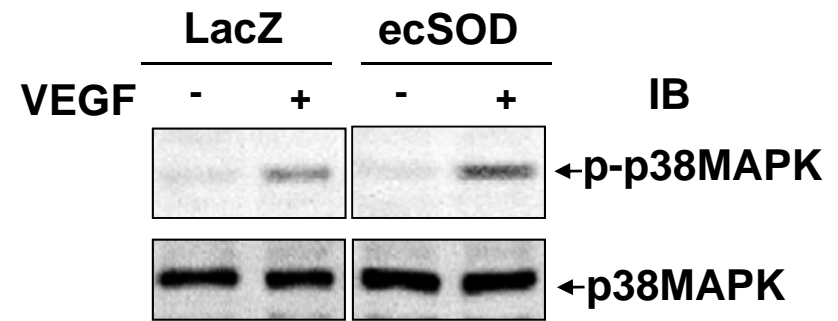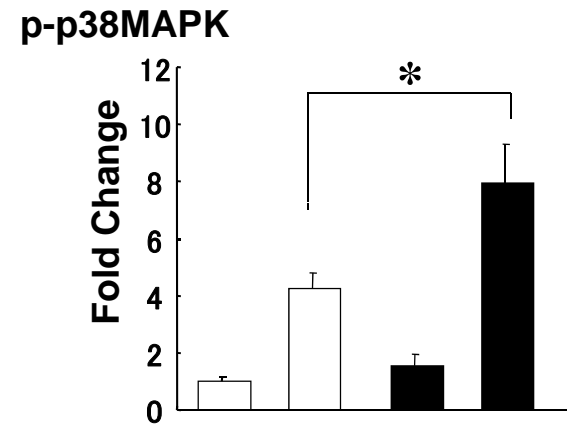

Supplement: Figure S7 — ecSOD enhances VEGFR2 downstream signaling in HUVECs. Cell lysates from Ad.LacZ and Ad.ecSOD infected HUVECs with or without VEGF stimulation (20 ng/ml, 5 min) were IB with anti-p-PLCγ or PLCγ (A) or p-p38MAPK or p38MAPK (B) antibodies (n = 3). *p<0.05 (0.08 MB PDF) [file pone.0010189.s007.pdf]
